# Inhaled nitric oxide in premature infants for preventing bronchopulmonary dysplasia: a meta-analysis

**DOI:** 10.1186/s12887-023-03923-4

**Published:** 2023-03-29

**Authors:** Yi Zheng, Qi Wu, Shuping Han

**Affiliations:** Department of Pediatrics, Women’s Hospital of Nanjing Medical University (Nanjing Maternity and Child Health Care Hospital), Nanjing, China

**Keywords:** Nitric oxide, Infant, Premature, Bronchopulmonary dysplasia, Meta-analysis

## Abstract

**Background:**

The effectiveness of nitric oxide (NO) in reducing the risk of bronchopulmonary dysplasia (BPD) remains debatable. In this study, we performed a meta-analysis to guide clinical decision-making regarding the significance of inhaled NO (iNO) on the potential occurrence and outcomes of BPD in premature infants.

**Methods:**

Data from clinical randomized controlled trials (RCTs) published in PubMed, Embase, Cochrane Library, Wanfang, China National Knowledge Infrastructure (CNKI) and Chinese Scientific Journal Database VIP databases for premature infants were searched from inception to March 2022. Review Manager 5.3 statistical software was used for heterogeneity analysis.

**Results:**

Of the 905 studies retrieved, 11 RCTs met the screening criteria of this study. Our analysis showed that the iNO group was associated with a significantly lower incidence of BPD than the control group (relative risk *[RR]* = 0.91, 95% confidence interval (*CI*) 0.85-0.97, *P* = 0.006). We also observed no significant difference in the incidence of BPD between the two groups at the initial dose of 5 ppm (ppm) (*P* = 0.09) but those treated with 10 ppm iNO had a significantly lower incidence of BPD (*RR* = 0.90, 95%*CI* 0.81-0.99, *P* = 0.03). However, it should be noted that although the iNO group had an increased risk for necrotizing enterocolitis (NEC) (*RR* = 1.33, 95%*CI* 1.04-1.71, *P* = 0.03), cases treated with an initial dose of 10 ppm revealed no significant difference in the incidence of NEC compared with the control group (*P* = 0.41), while those treated with an initial dosage of 5 ppm of iNO had a significantly greater NEC rates than the control group (*RR* = 1.41, 95%*CI* 1.03-1.91, *P* = 0.03). Further, we observed no statistically significant differences in the incidence of in-hospital mortality, intraventricular hemorrhage (IVH) (Grade 3/4) or periventricular leukomalacia (PVL) and pulmonary hemorrhage (PH) between the two treatment groups.

**Conclusions:**

This meta-analysis of RCTs showed that iNO at an initial dosage of 10 ppm seemed more effective in reducing the risk of BPD than conventional treatment and iNO at an initial dosage of 5 ppm in preterm infants at a gestational age of ≤34 weeks who required respiratory support. However, the incidence of in-hospital mortality and adverse events between the overall iNO group and Control were similar.

## Background

Bronchopulmonary dysplasia (BPD) is a severe respiratory condition that negatively impacts the survival of premature infants. The early survival rate of preterm infants has significantly improved with new developments in intensive care unit (ICU) technology. The prevalence of BPD in premature newborns at gestational age less than 28 weeks is reported to range from 48 to 68% [1]. The pathophysiology of BPD has shifted dramatically as a result of changes in epidemiology, clinical symptoms and prenatal and postnatal management of preterm infants. The “new” BPD is more frequent in premature newborns with a gestational age of less than 26 weeks and a birth weight of less than 1000 g [2]. Early non-invasive management of BPD includes the use of antenatal corticosteroids (ANS) to accelerate lung maturation in preterm birth from 24 weeks to 34 weeks of gestation [3], surfactant administration via a small catheter in spontaneously breathing infants to avoid mechanical respiratory support [4], inhaled bronchodilators to decrease airway resistance and wheezing and increase dynamic compliance [5], and many more [6]. However, the outcomes of current preventions for BPD remain unsatisfactory and should be further improved.

Nitric oxide (NO), a vasoactive chemical generated and released by vascular endothelial cells, selectively relaxes pulmonary vascular smooth muscle [7]. NO has selective diastolic effects on the pulmonary artery, which might minimize lung tissue oxidative damage and improve lung development, alveolarization and vascular remodeling [7]. The US Food and Drug Administration has approved inhaled NO (iNO) for the treatment of neonates aged ≥34 weeks with hypoxemic respiratory failure (HRF) and persistent pulmonary hypertension (PPHN) [8].

However, because iNO treatment might produce major adverse events such as oxidative stress damage and bleeding, the National Institutes of Health does not recommend it in premature infants [9]. Peluso et al. conducted a population-based cross-sectional research on premature infants at gestational age less than 35 weeks and found that iNO use increased from 2011 to 2016 compared to 2004 to 2010 [10]. Currently, there are disagreements regarding patient selection, age of inclusion, initial and maximum dose, course of treatment and efficacy on the usage of iNO. In this study, we performed a systematic review and meta-analysis to comprehensively and objectively evaluate the efficacy and safety of iNO in the prevention of BPD to aid clinical decision-making.

## Methods

### Literature search and selection criteria

We conducted a literature search on PubMed, EMBASE and the Cochrane Library from the databases’ inception to March 2022 using keywords “bronchopulmonary dysplasia” or “dysplasia, bronchopulmonary” combined with the terms “infant, premature”, “infants, premature”, “premature infant”, “preterm infants”, “infant, preterm”, “infants, preterm”, “preterm infant”, “premature infants”, “neonatal prematurity”, “prematurity, neonatal”, and “nitric oxide”, “oxide, nitric”, “nitrogen monoxide”, “monoxide, nitrogen”, “nitric oxide, endothelium-derived”, “endothelium-derived nitric oxide”, “nitric oxide, endothelium-derived”, “endogenous nitrate vasodilator”, “nitrate vasodilator, endogenous”, “vasodilator, endogenous nitrate”, “mononitrogen monoxide”, “monoxide, mononitrogen”. The languages used in the search were in Chinese and English. We also searched literature from the Wanfang, China National Knowledge Infrastructure (CNKI) and Chinese Scientific Journal Database VIP databases using Chinese terminologies of the same keywords and time period.

Using the above-mentioned search approach, two investigators independently screened the titles, abstracts and full texts of potentially eligible studies. Literature meeting the following criteria were included: (1) randomized controlled trials (RCTs); (2) involved premature infants at a gestational age ≤ 34 weeks who had HRF and received respiratory assistance; and (3) compared iNO to an inhalation placebo. Overall, the iNO group comprised premature infants who underwent conventional measure plus iNO, whereas the control group underwent conventional measure plus inhalation placebo. Studies were excluded if they: (1) were case reports, literature reviews, conference abstracts or systematic evaluations, (2) contained retrospective, non-random or incorrect random methods literature, (3) included animal experiments, (4) were repeated publication of research literature, and (5) did not have complete data of interest for this study or full text.

### Data abstraction

Two investigators worked independently on the literature screening, data extraction and literature quality rating. Disagreements were handled by a third researcher. The last name of the first author, year of publication, sample size, age of enrollment, iNO start and maximum dose, duration of iNO, primary endpoints and diagnostic criteria were all collected from eligible papers using a predesigned excel form. Induced in-hospital mortality and BPD incidence were the key outcome metrics. Treatment safety data, based on the incidence of intraventricular hemorrhage (IVH) (Grade 3/4) or periventricular leukomalacia (PVL), pulmonary hemorrhage (PH) and necrotizing enterocolitis (NEC), were collected as secondary outcomes of measures, and subgroup analysis was performed on the initial iNO treatment dose of 5 ppm (ppm) and ≥ 10 ppm. In addition, the diagnosis of BPD was based on “classical” BPD criteria [11–13] in 5 studies [14–18], “new” BPD criteria [19, 20] in 5 studies [21–25].

### Quality assessment

Two independent reviewers assessed the study designs. We used the Cochrane Systematic Review Manual to evaluate the quality of the observational studies based on the following six sections [26]: random allocation method; hidden allocation scheme; blind method to research objects, researchers and statisticians of research results; integrity of result data; publication bias; and other sources of bias. In the statistical process, the quality assessment was classified as follows. All indexes with low risk were evaluated as having low bias risk and the literature was of high quality; studies with more than 1 item of uncertainty risk were assessed as the risk of uncertainty bias, and studies with more than 1 item of high risk were assessed as the risk of high bias.

### Statistical analysis

The Review Manager 5.3 software was used to conduct the meta-analyses. Heterogeneity was assessed using the *Chi-square* test. The fixed-effect model was adopted for analysis since *I*^*2*^ ≤ 50% and *P* ≥ 0.1 suggested statistical homogeneity between studies. The random-effect model was employed to analyze studies with *I*^*2*^ > 50% or *P* < 0.1. Dichotomous variables were analyzed using relative risk *[RR]* with 95% confidence interval (*CI*). Funnel plot was used to assess the risk of publication bias. *P* < 0.05 was used to determine statistical significance.

## Results

### Literature retrieval and basic features

**Fig. 1:**
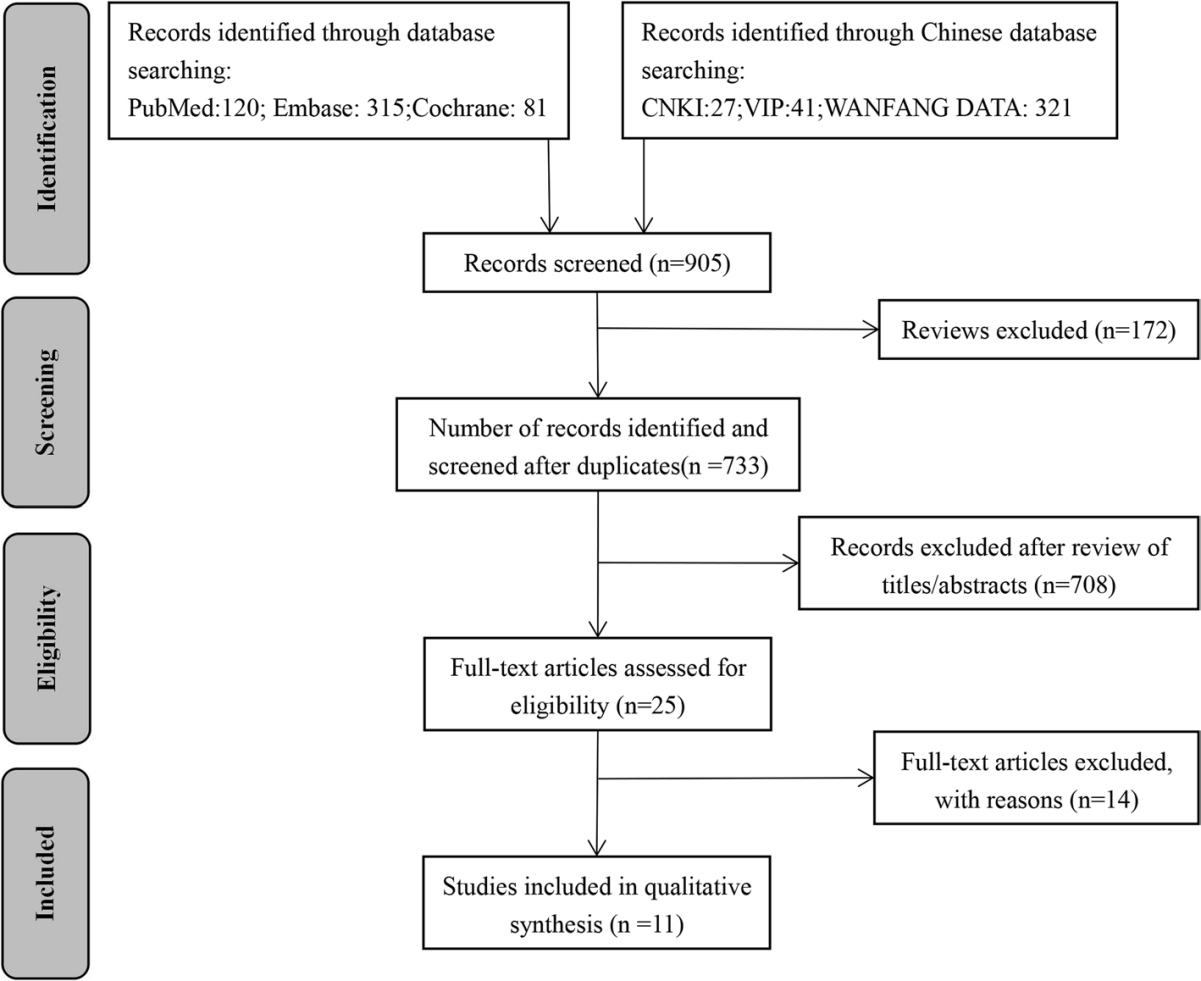
Flowchart for the systematic literature research

A total of 905 studies were initially retrieved. Following an initial screening, 172 studies were excluded due to duplication and after screening the title and abstract of the remaining records, 708 studies were further excluded. Following full-text assessments of the remaining studies, 11 RCTs were found eligible for this study, comprising 10 English and 1 Chinese literature [14–18, 21–25, 27]. There were 3651 preterm infants in all. Figure 1 illustrates the flow chart of literature retrieval, and Table 1 shows the characteristics of included research.

**Table 1:** Characteristics of the included studies in the meta-analysis

First author (Year)	Origin	Sites,n	Groups (T/C)	GA,wk	BW,g	Age at Enrollment	start/max iNO,ppm	Duration of iNO,d	Primary endpoints	Diagnostic criteria
Ballard 2006 [14]	USA	21	294/288	≤32	500-1250	7-21d	20/20	≥24	death or BPD at 36 weeks	Walsh 2004
Dani 2006 [15]	Italy	1	20/20	<30	T:937 ± 298^a^ C:825 ± 299.3^a^	<7d	10/10	4.1^b^	death or BPD at 36 weeks	Shennan 1988
Hasan 2017 [21]	Canada	33	229/222	<30	<1250	5-14d	20/20	24	survival without BPD	NICHD
Hascoet 2005 [27]	France, Belgium	10	61/84	<32	–	6-48 h	5/10	–	survival at 28 days of age	–
Kinsella 2006 [16]	USA	16	398/395	≤34	500-1250	<48 h	5/5	21	death or BPD at 36 weeks	Toce 1984
Kinsella 2014 [22]	USA	5	59/65	≤34	500-1250	<72 h	10/10	≥14	death or BPD at 36 weeks	NICHD
Mercier 2010 [23]	EUNO	36	399/401	24-28	>500	<24 h	5/5	7-21	survival without BPD	NICHD
Schreiber 2003 [17]	USA	1	105/102	<34	<2000	<72 h	10/10	7	death or CLD	Shennan 1988
Van Meurs 2005 [24]	USA	16	210/210	<34	401-1500	24 h	5/10	≤14	death or BPD at 36 weeks	NICHD
Van Meurs 2007 [18]	USA	16	14/15	<34	>1500	24 h	5/10	≤14	death or BPD at 36 weeks	Walsh 2004
Wei 2014 [25]	China	1	30/30	26-34	1082-2350	<7d	5/10	≥7 or until the ventilator is removed	death or BPD at 36 weeks	NICHD

-Not described in the article

^a^Mean ± SD

^b^Median

### Quality assessment

**Fig. 2:**
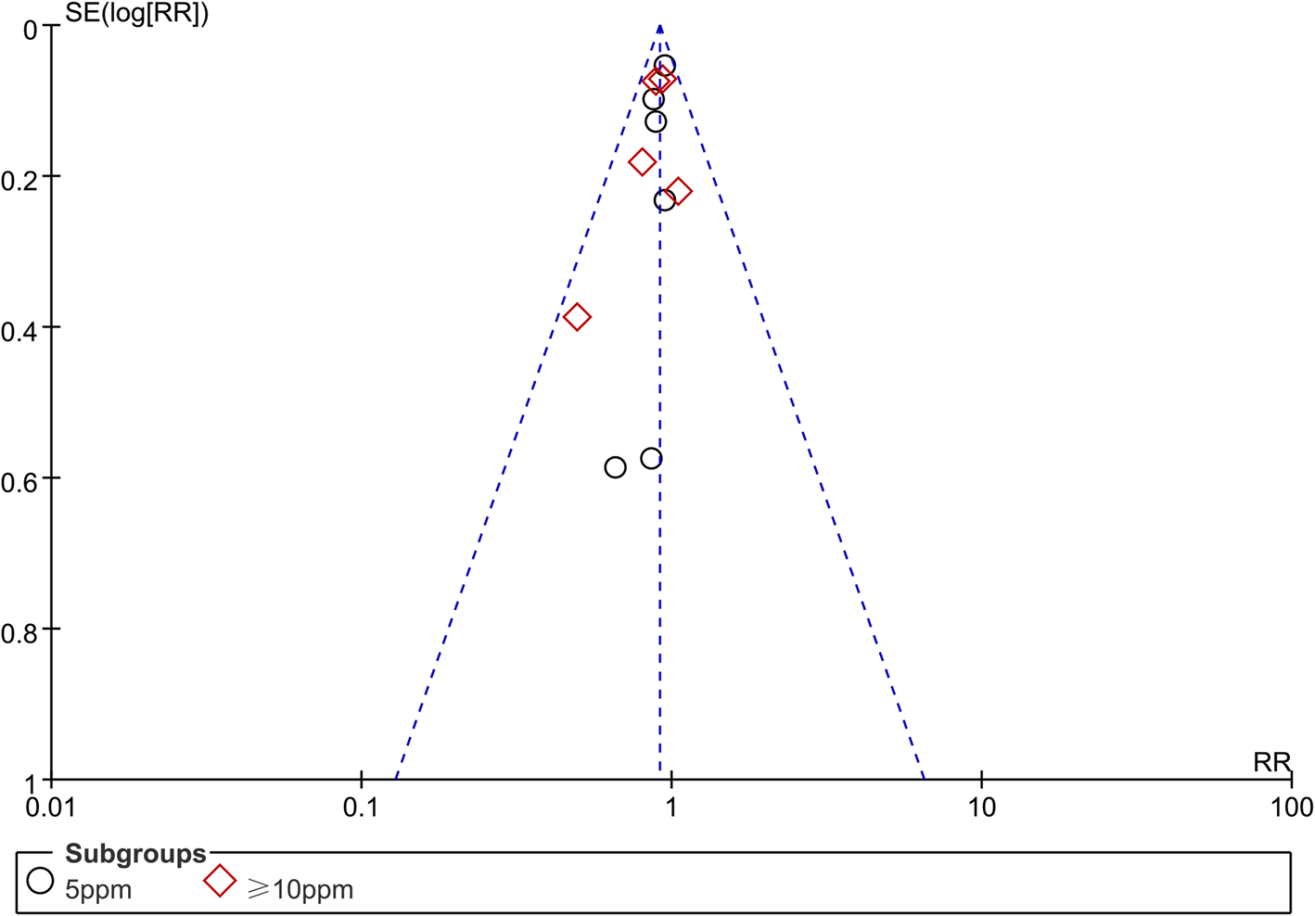
Funnel plot of 11 RCTs

Among the 11 RCT studies included in this study, 7 had a low risk of bias [14, 17, 18, 21–24], 2 had an uncertain risk of bias [16, 25], while the remaining 2 had a high risk of bias [15, 27]. Funnel plot results showed that most of the studies were located at the upper part of the funnel without obvious asymmetry, suggesting that there was no significant publication bias (Fig. 2).

### Main outcome measures

#### Rate of in-hospital mortality

**Fig. 3:**
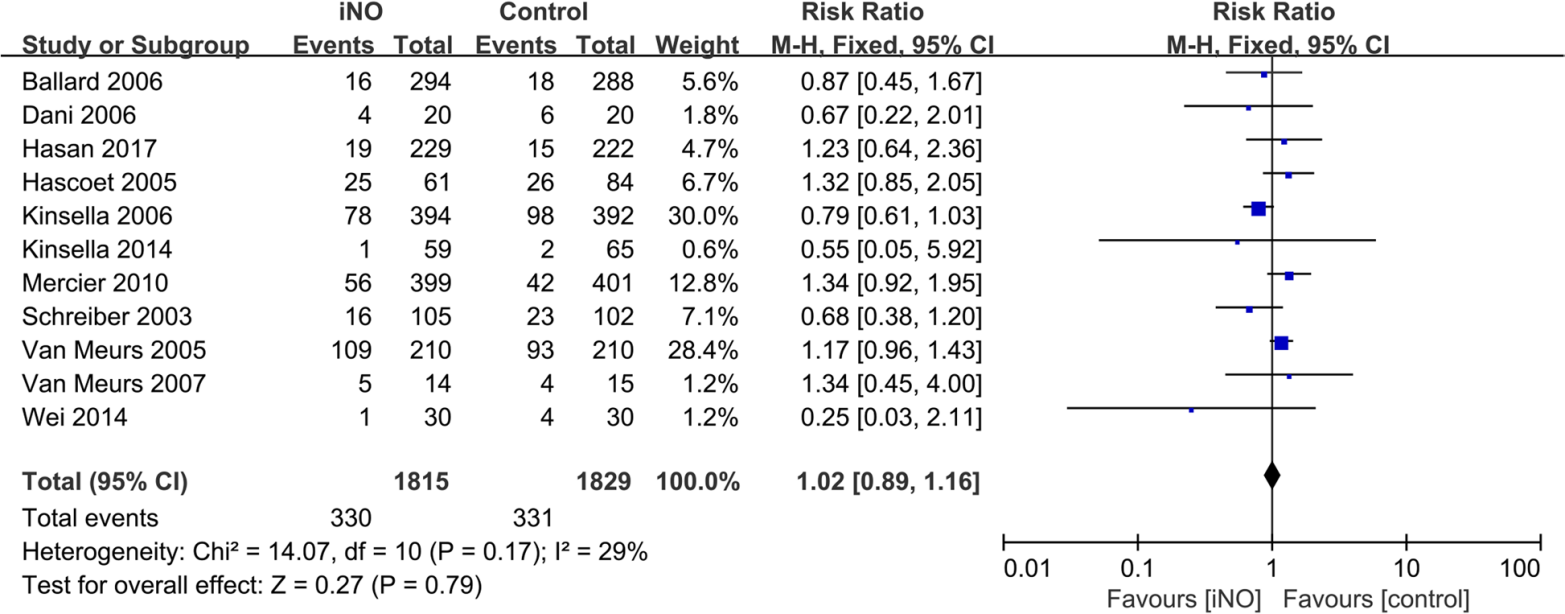
Efficacy of iNO versus Control on in-hospital mortality

Eleven studies evaluated the effects of iNO versus Control on the in-hospital mortality of premature infants [14–18, 21–25, 27]. The fixed-effect model was employed because the heterogeneity test revealed that *I*^*2*^ = 29% and *P* = 0.17. The meta-analysis results showed no statistically significant difference in the in-hospital mortality rates between the iNO and control groups (*RR* = 1.02, 95% *CI* 0.89-1.16, *P* = 0.79, Fig. 3).

#### Rate of BPD

**Fig. 4:**
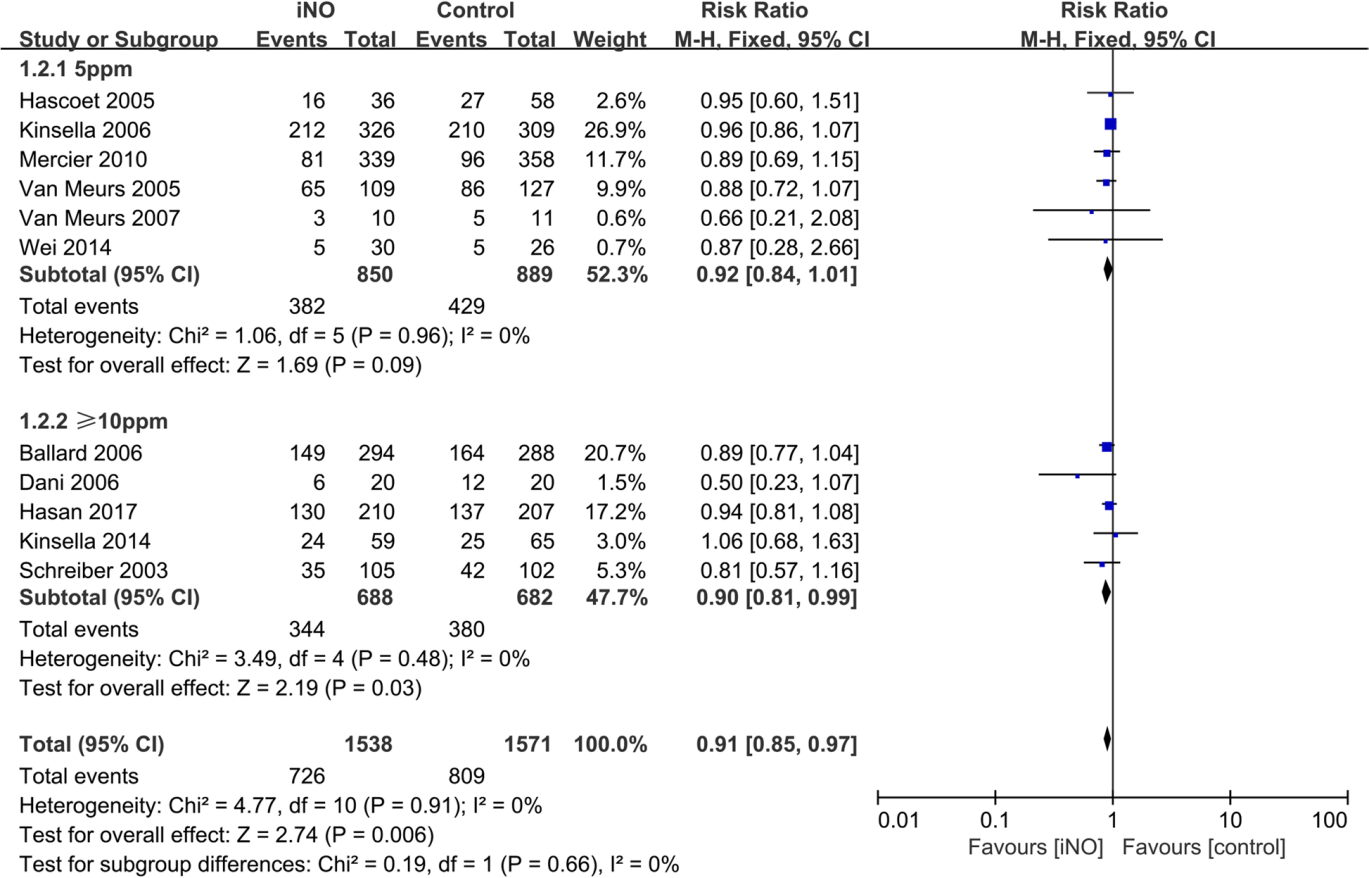
Efficacy of iNO versus Control in reducing the incidence of BPD

Eleven studies [14–18, 21–25, 27] examined the prevalence of BPD in the iNO and control groups. The fixed-effect model was employed as the heterogeneity test revealed that *I*^*2*^ = 0% and *P* = 0.91. The meta-analysis results revealed a significant difference in the rate of BPD between the two groups (*RR* = 0.91, 95% *CI* 0.85-0.97, *P* = 0.006), with iNO being significantly superior to Control in reducing the incidence of BPD. Subgroup analysis with varied initial treatment doses revealed no significant difference in the incidence of BPD between the iNO group and the control group at the initial dose of 5 ppm (*RR* = 0.92, 95% *CI* 0.84-1.01, *P* = 0.09). However, for a dose of ≥10 ppm, iNO was associated with a lower incidence of BPD compared with the control group (*RR* = 0.90, 95% *CI* 0.81-0.99, *P* = 0.03, Fig. 4).

### Secondary outcome measures

#### Rate of IVH (grade 3/4) or PVL

**Fig. 5:**
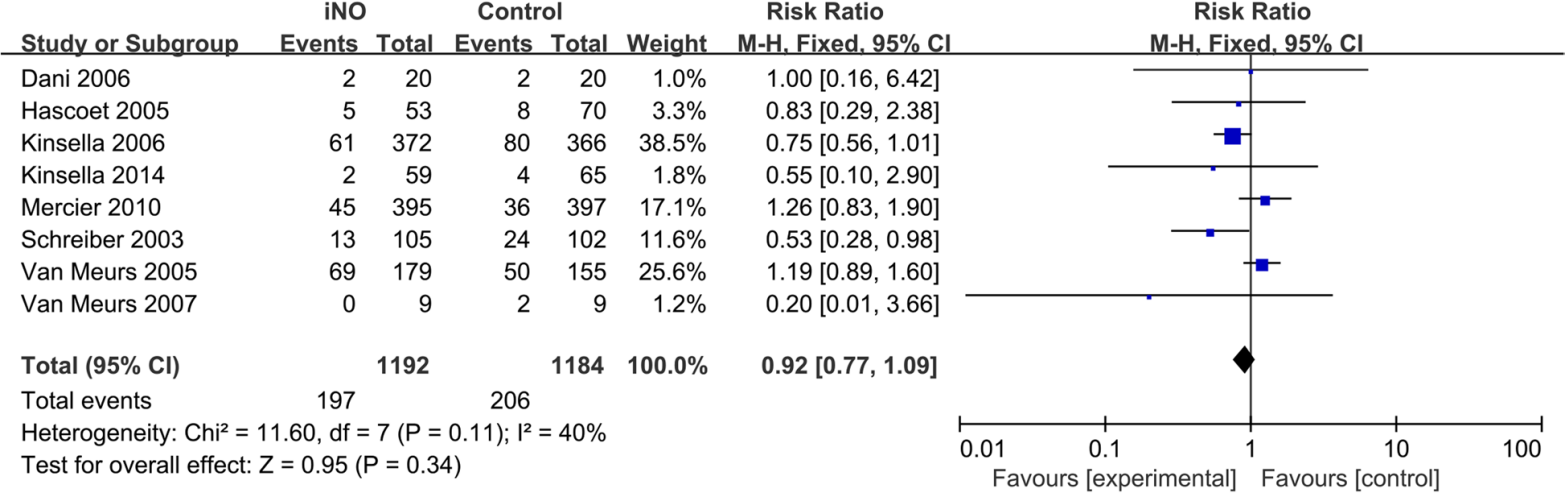
Efficacy of iNO versus Control on IVH (Grade 3/4) or PVL

Eight studies [15–18, 22–24, 27] documented the incidence of IVH (Grade 3/4) or PVL. The fixed-effect model was employed as the heterogeneity test revealed that *I*^*2*^ = 40% and *P* = 0.11. The results revealed no significant difference in the incidence of IVH (Grade 3/4) or PVL between the iNO group and the control group (*RR* = 0.92, 95% *CI* 0.77-1.09, *P* = 0.34, Fig. 5).

#### Rate of PH

**Fig. 6:**
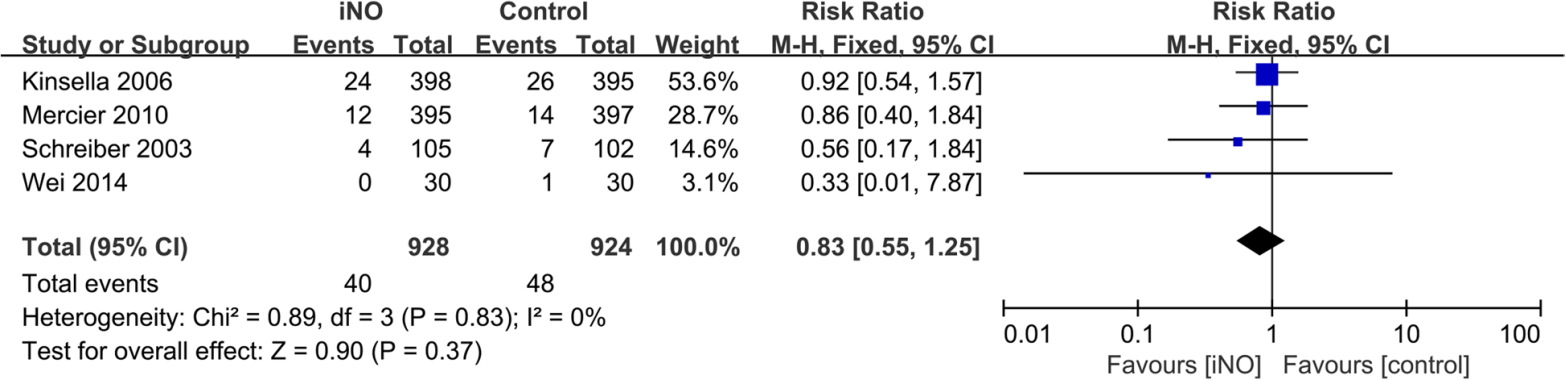
Efficacy of iNO versus Control on PH

Four investigations [16, 17, 23, 25] examined the prevalence of PH in the iNO and control groups. The fixed-effect model was employed because the heterogeneity test revealed that *I*^*2*^ = 0% and *P* = 0.83. The results of the meta-analysis revealed no significant difference in PH rate between the iNO and control groups (*RR* = 0.83, 95% *CI* 0.55-1.25, *P* = 0.37, Fig. 6).

#### Rate of NEC

**Fig. 7:**
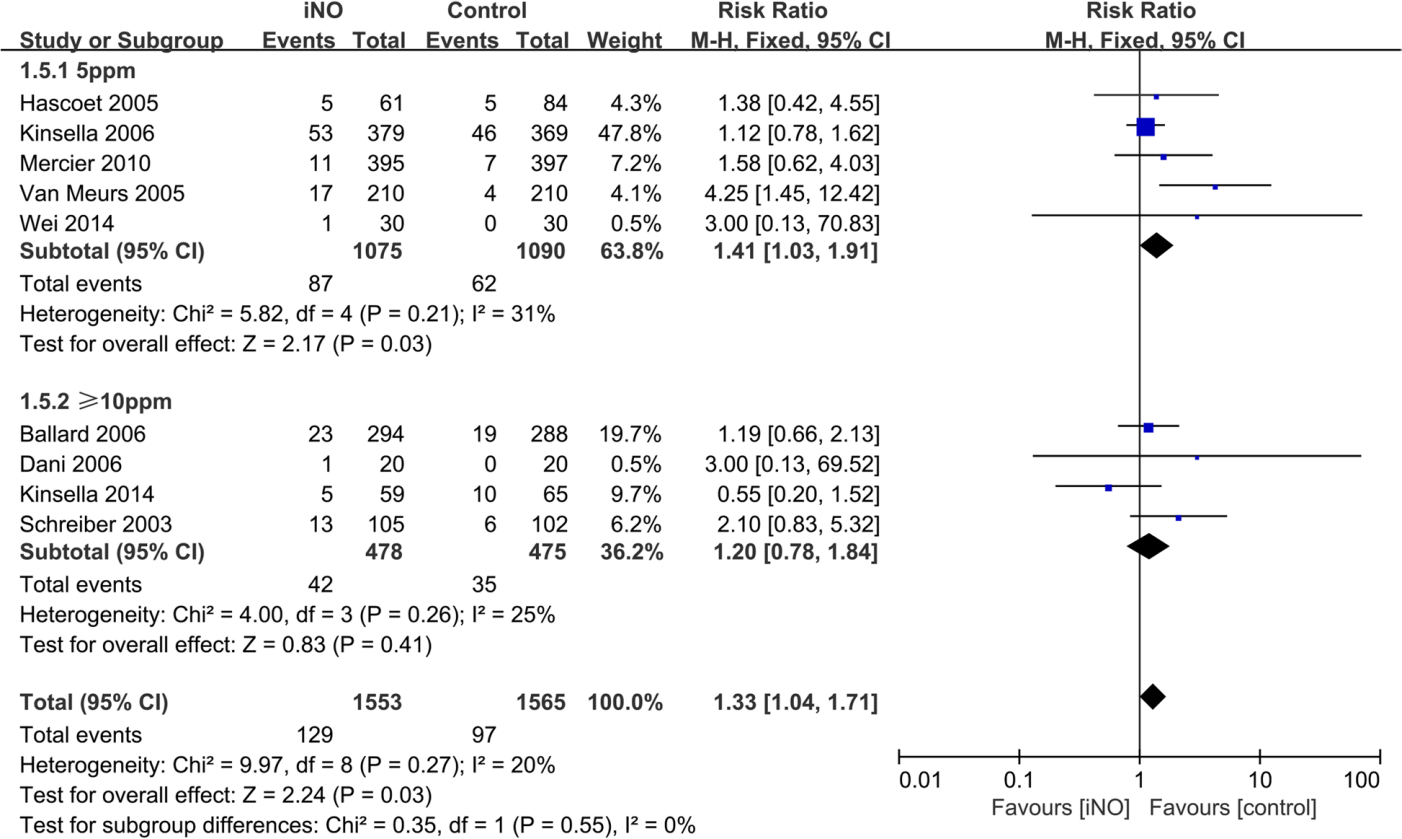
Efficacy of iNO versus Control on NEC

Nine studies [14–17, 22–25, 27] assessed the prevalence of NEC in the iNO and control groups. The fixed-effect model was employed as the heterogeneity test revealed that *I*^*2*^ = 20% and *P* = 0.27. The meta-analysis results revealed a significant difference in NEC rate between the two groups (*RR* = 1.33, 95% *CI* 1.04-1.71, *P* = 0.03), indicating that iNO was associated with an increase in NEC incidence compared with the control group. However, iNO at an initial dose of ≥10 ppm had similar incidence of NEC to the control group (*RR* = 1.20, 95% *CI* 0.78-1.84, *P* = 0.41), while iNO at the initial dose of 5 ppm was associated with a significantly higher incidence of NEC than the control group (*RR* = 1.41, 95% *CI* 1.03-1.91, *P* = 0.03, Fig. 7).

## Discussion

In conclusion, the meta-analysis results revealed a reduced incidence rate of BPD following the iNO measure but no statistically significant difference in in-hospital mortality versus the control group. Even though the designs of the available RCTs for the early use of iNO in preterm newborns varied, we did not observe a significant reduction in mortality and the differences were not statistically significant. Moreover, BPD incidence decreased by 10% (*P* = 0.03) when the initial dose of iNO was ≥10 ppm compared to the control group. Due to the varied methodologies and oxygenation indicators investigated in each included study, a direct meta-analysis could not be conducted. However, based on the study’s findings, we hypothesize that iNO decreased the incidence of BPD because it could increase oxygenation. Although multiple factors may influence BPD, inflammation is one of its most critical primary causes [28]. Earlier studies suggested that low NO levels could reduce lung inflammation [29], whereas subsequent research revealed that high NO levels could also exacerbate lung inflammation [30]. iNO may alleviate lung inflammation by reducing the incidence of BPD; however, the incidence of BPD could not be reduced significantly due to the dual effect of NO and contradictory results obtained from different tests. Additional research is required to determine whether pulmonary inflammation causes BPD and whether there are changes in inflammatory conditions, such as inflammatory factor levels. In this study, we also investigated the potential adverse effects of iNO based on the incidence of IVH (Grade 3/4) or PVL, PH, and NEC. INO had no statistically significant association with the incidence of IVH (Grade 3/4) or PVL and PH, but it may have increased the risk of NEC (subgroup analysis, iNO at an initial dose of 5 ppm versus control, *P* = 0.03; Fig. 7).

The results of nine studies comparing the incidence of NEC in the iNO group and the control group indicated that the incidence of NEC was 30% higher in the iNO group than in the control group, indicating that iNO may increase the risk of NEC, which was consistent with the findings of Van Meurs et al. [24]. Mercier et al. [23] reported a correlation between the induction of NEC by iNO and the gestational age of premature infants, which requires further investigation in future studies. The initial dose of ≥10 ppm was not statistically significant (*P* = 0.41) based on subgroup analysis employing varying initial treatment dosages. Askie et al. [31] also reported that a higher starting dose might be associated with improved HRF treatment outcomes in premature infants. In addition, the incidence of NEC in the iNO group was nearly 40% higher than in the control group (*P* = 0.03), suggesting that iNO may increase the risk of NEC in preterm newborns at an initial dose of 5 ppm. According to a 2016 review [32], excessive nitric oxide synthase (NOS) expression in the intestinal tract with high NO or superoxide nitrite levels led to epithelial cell loss and microbial infection, which resulted in NEC. Further research is necessary to determine whether iNO could increase the expression of NOS in the intestinal tract or result in excessive NO or superoxide nitrite production. In a clinical trial conducted in 2012 [33], iNO therapy was found to increase nitrite and nitrate levels in whole blood by approximately twofold, possibly due to a reduction in the oxygen-carrying capacity of blood cells in preterm newborns, thereby inducing NEC due to hypoxia in the digestive tract.

Although we demonstrated the potential effects of different doses of iNO on preventing the occurrence of BPD, the survival outcome was not significantly higher than with conventional treatment. Other potential alternatives to reduce the risk of BPD occurrence include early vitamin D, postnatal corticosteroids, and antibiotics [34–36]. Ge et al. found that early vitamin D supplementation (800 IU/day within 48 hours of birth for 28 days) could significantly reduce the incidence of BPD in premature infants [34]. According to Ramaswamy et al., an early, cumulative dose of systemic dexamethasone may be the best regimen for preventing mortality or BPD at 36 weeks postmenstrual age among 14 regimens from 62 studies [36]. Ozdemir et al. [35] concluded that clarithromycin effectively prevented BPD in preterm infants weighing between 750 and 1200 g and colonized with *Ureaplasma urealyticum*. Comparatively, we found in this study that the initial dose of iNO may be associated with BPD and NEC, which has important clinical implications for iNO prevention of BPD.

Several limitations were present in this study. (1) The “classical” and “new” forms of BPD have distinct diagnostic criteria. The “classical” BPD is based on RDS and emphasizes oxygen dependence and lung damage demonstrated by clinical and imaging evidence without grading the severity of BPD. Therefore, clinical criteria must be updated to evaluate the severity and long-term prognosis of BPD. (2) The limited overall sample size and the small number of included research samples affect the reliability of this study to some degree. (3) There was no subgroup analysis on birth weight, the start time of inhalation, and iNO inhalation dose range of preterm infants, which affected the reliability of this study. For optimal iNO clinical guidance, further research is required to clarify the clinical significance of iNO based on neonatal birth weight, persistent period, and inhalation range.

## Conclusions

This review of RCTs on preterm infants at a gestational age of ≤34 weeks who require respiratory support indicates that iNO at an initial dosage of 10 ppm appeared more effective in reducing the risk of BPD than conventional treatment. In comparison, iNO at an initial dosage of 5 ppm had a comparable incidence of in-hospital mortality and adverse events compared with conventional treatment plus placebo. More research is required to improve the in-hospital mortality and safety of iNO in this setting.

## Data Availability

All data used during the study are available from the corresponding author by request.

## Abbreviations

NO: Nitric oxide
BPD: Bronchopulmonary dysplasia
iNO: Inhaled NO
RCTs: Randomized controlled trials
CNKI: China National Knowledge Infrastructure
RR: Relative risk
CI: Confidence interval
ppm: Parts per million
NEC: Necrotizing enterocolitis
IVH: Intraventricular hemorrhage
PVL: Periventricular leukomalacia
PH: Pulmonary hemorrhage
ICU: Intensive care unit
ANS: Antenatal corticosteroids
HRF: Hypoxemic respiratory failure
PPHN: Persistent pulmonary hypertension
NOS: Nitric oxide synthase
